# A Comparative Study of RQA-Guided Attention Mechanisms with LSTM Autoencoder for Bearing Anomaly Detection

**DOI:** 10.3390/s26031015

**Published:** 2026-02-04

**Authors:** Ayşenur Hatipoğlu, Ersen Yılmaz

**Affiliations:** 1 Electrical & Electronics Engineering Department, Bursa Uludağ University, 16059 Bursa, Türkiye; ersen@uludag.edu.tr; 2 Turkish Aerospace Industries Inc., Uludağ University R&D Center, 16059 Bursa, Türkiye

**Keywords:** anomaly detection, LSTM autoencoder, recurrence quantification analysis, attention mechanism, vibration signals, time-series analysis

## Abstract

Accurate anomaly detection in rotating machinery under noisy conditions remains challenging in Prognostics and Health Management (PHM). Existing deep learning autoencoders and attention mechanisms rely primarily on data-driven similarity measures and fail to explicitly incorporate nonlinear dynamical characteristics of degradation. In this study, we propose a Recurrence Quantification Analysis-Aware Attention (RQAA) framework that systematically injects chaos-theoretic descriptors into the attention mechanism of LSTM-based autoencoders for unsupervised anomaly detection. Specifically, RQA metrics including recurrence rate, determinism, laminarity, entropy, and trapping time are computed at the window level and embedded into the query-key-value attention scoring to guide the model toward dynamically informative temporal patterns. Three attention variants are developed to investigate different fusion strategies between learned representations and RQA-driven structural cues. The proposed framework is evaluated on three widely used bearing vibration datasets, which are IMS, CWRU, and HUST. Experimental results demonstrate that RQAA consistently outperforms conventional LSTM autoencoders and classical attention-based models, achieving up to 99.85% F1-score and 99.00% AUC while exhibiting superior robustness in low signal-to-noise scenarios. Further analysis reveals that explicit dynamical guidance enhances anomaly separability and reduces false alarms, particularly in early-stage fault detection. These findings indicate that integrating nonlinear dynamical information directly into attention scoring offers a principled and effective pathway for advancing unsupervised anomaly detection in rotating machinery and safety-critical industrial systems.

## 1. Introduction

Anomaly detection in PHM refers to the automated identification of deviations from expected system behavior and is essential for enabling proactive maintenance and preventing unexpected failures. Early approaches primarily relied on rule-based systems and handcrafted indicators. They were later replaced by data-driven machine learning techniques such as Support Vector Machine (SVM), Random Forests, K-Nearest Neighbor (KNN) and Principal Component Analysis (PCA) as multivariate sensor data became more widely available [1,2,3,4]. In recent years, deep learning models including Autoencoders (AE), Long-Short Term Memory (LSTM), and Gated Recurrent Unit (GRU) networks have gained prominence due to their ability to capture complex nonlinear and temporal dependencies in time-series data [5,6]. However, despite their strong performance, many existing approaches remain sensitive to operating condition variability and offer limited interpretability, motivating the development of more robust and structurally informed anomaly detection frameworks. Anomaly detection can be formulated as either a supervised or unsupervised learning task. While supervised approaches rely on labeled samples from both normal and faulty conditions, such annotations are often scarce, costly, or unreliable in complex engineering systems. As a result, anomaly detection in PHM is predominantly addressed in an unsupervised setting in which models are trained on healthy data to characterize normal operating behavior and identify deviations from it [6]. This formulation places strong emphasis on learning robust representations of normal dynamics that remain sensitive to subtle and early-stage degradations.

Recent advances in deep learning have enabled data-driven diagnostics for rotating machinery; however, widely adopted sequence modeling techniques, including Transformer-based self-attention, may struggle under noisy, non-stationary, or weakly separable operating conditions. Recurrence Quantification Analysis (RQA) provides a nonlinear dynamical framework for characterizing chaotic and recurrent structures in time-series signals [7]. In the existing literature, RQA has predominantly been employed as an offline feature extraction tool within hybrid learning pipelines [8].

While several recent studies have explored recurrence-based features and attention mechanisms for time series analysis, our approach differs fundamentally in its integration strategy. Recent work using recurrence plot images as CNN inputs for time series classification employs spatial feature extraction from RP visualizations, whereas our method computes scalar RQA metrics to directly modulate attention weights in LSTM autoencoders, preserving sequential information while injecting dynamical structure [9]. Similarly, multi-scale asymmetric recurrence plot approaches combined with Swin Transformers for bearing fault diagnosis apply vision transformers to RP images, treating the problem as spatial image processing rather than temporal sequence modeling [10]. MTF-based methods with mixed attention residual networks convert signals to 2D Markov transition field images and apply spatial attention, fundamentally differing from our approach which computes RQA metrics directly from time series and embeds them into temporal attention scoring for LSTM autoencoders [11]. Although physics-informed attention mechanisms have shown promise in PDE solving through PINNsFormer [12], where physical laws guide network learning, our work extends this paradigm to industrial anomaly detection by embedding chaos-theoretic RQA descriptors into attention scoring rather than differential equation constraints, thereby bridging physics-informed deep learning with practical PHM applications.

To the best of our knowledge, no prior study has systematically integrated RQA-derived nonlinear dynamical descriptors directly into the attention scoring mechanism of LSTM-based autoencoders for unsupervised bearing anomaly detection. Embedding RQA-informed structural cues into attention computation therefore offers a principled means of guiding attention toward dynamically informative regions of the signal.

The main contributions of this study are summarized as follows:

1. We propose three distinct RQA-enhanced attention mechanisms, namely Hybrid QKVRQAA, Input-level RQA-Guided Channel Attention (CRQAA), and Encoder-level RQA-Guided Channel Attention (ERQAA), which differ in how and where RQA-derived nonlinear dynamical information is mathematically integrated into the attention pipeline.
2. We provide a systematic comparative analysis within individual datasets demonstrating how different RQA integration levels influence representation learning and anomaly detection performance under varying signal dynamical regimes, including non-stationary and noisy conditions.
3. Extensive experiments conducted on three publicly available bearing datasets (IMS, CWRU, and HUST) show that embedding RQA-derived dynamical descriptors into attention mechanisms consistently improves anomaly detection performance, particularly for signals exhibiting nonlinear and chaotic characteristics.
4. By computing RQA metrics directly from raw vibration signals, the proposed approach incorporates physics-informed dynamical priors into deep attention models, effectively bridging nonlinear dynamical system analysis and data-driven representation learning.

The remainder of this manuscript is organized to first review related work, then present the proposed methodology, followed by experimental evaluation, and finally conclude with directions for future research.

## 2. Related Work

**Figure 1:**
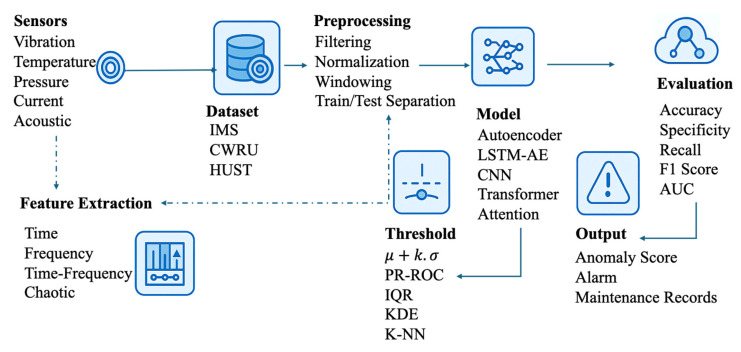
Anomaly detection application general diagram.

PHM systems are typically implemented through a structured processing pipeline encompassing data acquisition, preprocessing, representation learning, anomaly scoring, and decision support. Although specific modeling techniques vary across applications, most anomaly detection frameworks follow a common workflow that integrates signal processing, feature learning, and decision-making components. Figure 1 illustrates this general anomaly detection pipeline, which provides a contextual framework for positioning the methods reviewed in this section as well as the approach proposed later in the paper.

According to the literature, signal-, model-, or data-based methods, as well as deep learning and hybrid approaches, are widely used in anomaly detection [13]. Examples of signal-based methods include FFT, WT, and RQA [14,15]. Within model-based methods, state-space modeling and KF are frequently mentioned [16]. Data-based machine learning methods include approaches such as Isolation Forests (IF), Principal Component Analysis (PCA), Single-Class SVM, and K-Means clustering. On the deep learning side, AE, LSTM-AE, CNNs, and attention-based models are reported to be used in anomaly detection [17]. Furthermore, hybrid approaches combining attention-based mechanisms with data-driven methods have also been reported [18]. Attention mechanisms enable the model to focus on critical time steps or sensor channels, allowing for the suppression of unnecessary information and more effective learning of long-term dependencies [19]. Different types of attention have been proposed in the literature [20]. Channel attention mechanisms are used to highlight important feature channels, particularly in convolutional architectures, while spatial and temporal attention mechanisms highlight critical regions in the input space and important time steps in sequential data, respectively. Multi-headed attention can capture different types of relationships by processing the input in parallel attention subspaces, while external attention mechanisms aim to increase computational efficiency through external memory structures [21,22,23]. Self-attention mechanisms are widely used in time series modeling tasks due to their ability to selectively focus on the most informative parts of the sequence [24]. This structure provides an advantage, particularly in capturing slow-developing decay trends and long-term dependencies [25]. Compared to recurrent models, its ability to process the entire sequence simultaneously increases computational efficiency and facilitates adaptation to variable and noisy working conditions. Furthermore, attention weights explicitly reveal which time steps the model considers more important in its decision-making process, thereby supporting interpretability. When combined with LSTM architectures, attention operates as a temporal weighting mechanism that highlights informative segments of sequential data, whereas CNN-based models emphasize salient patterns across sensor channels. In contrast, Transformer architectures employ self-attention to jointly capture long-range temporal dependencies and global inter-sensor interactions [24].

Many engineering systems, particularly rotating machinery, exhibit nonlinear dynamic behavior in which the system output is not directly proportional to its input and cannot be adequately described using linear equations. In rotating machines, nonlinear events arising from mechanical interactions, wear processes, and operating variability motivate the use of chaos-based analysis techniques for vibration signal interpretation. Chaotic analysis enables the characterization of nonlinear system dynamics, supports prediction and forecasting, facilitates anomaly detection, and provides insight into system stability and degradation mechanisms. Within this context, RQA has emerged as an effective tool for analyzing nonlinear and non-stationary time series.

Compared to traditional time-frequency methods such as Fourier Transform or Wavelet Analysis, RQA offers distinct advantages for nonlinear and non-stationary signals. RQA does not assume stationarity, requires relatively short time series, and can detect subtle changes in system dynamics that may not be apparent in spectral analysis [26]. These characteristics make RQA particularly suitable for condition monitoring of rotating machinery, where transient events and nonlinear dynamics are prevalent.

RQA has proven valuable for assessing signal instability, which is a common property of real-world vibration data [27,28]. By extracting sensitive recurrence-based features, RQA-based methods improve the interpretability and robustness of fault diagnosis even under noisy operating conditions [29]. Moreover, the instability of recurrence quantification measures has demonstrated strong predictive capability when characterizing complex dynamical behavior [30]. Owing to their robustness to noise, ability to extract comprehensive dynamical features, and computational efficiency, RQA-based approaches are suitable for both exploratory analysis and real-time monitoring applications [28,29]. Consequently, RQA has been applied in a wide range of PHM tasks, including early detection of aircraft engine failures, identification of bearing and rotor faults via vibration analysis, RUL estimation in chaotic systems, and time series analysis of helicopter and aircraft sensor data [7,8].

In the literature, RQA has been applied to monitor transient accelerometer signals from auxiliary aircraft equipment such as fuel pumps, where it provides early warnings of degradation and improves mean time before failure estimation. Studies have shown that combining RQA with traditional diagnostic methods can enhance failure detection accuracy and maintenance planning for aircraft components, outperforming classical models such as k-Nearest Neighbor and Random Forest, and demonstrating strong potential for engineering applications, including aviation bearings [7,31]. Furthermore, the integration of RQA with Kalman filtering techniques has enabled the prediction of bearing failures by extracting entropy-based features from vibration signals and modeling degradation dynamics, with reported prediction horizons of up to 50 min prior to failure [32]. Beyond mechanical systems, RQA has also been used to analyze surface pressure data on wing profiles, successfully distinguishing flow transitions at different angles of attack, and to interpret turbulence measurements by separating turbulent and non-turbulent segments using recurrence-based variables, thereby reducing subjectivity in boundary definitions [33].

Despite the extensive body of work on anomaly detection in PHM, most existing studies employ nonlinear dynamical descriptors such as RQA either as standalone diagnostic indicators or as offline feature extraction tools integrated into conventional machine learning pipelines. In parallel, attention mechanisms in deep learning models are predominantly driven by data similarity measures and temporal correlations, which can be sensitive to noise and non-stationary operating conditions. As a result, the structural dynamical information captured by recurrence-based analysis remains largely untapped within attention scoring mechanisms. This gap motivates the present study, which systematically integrates RQA-derived nonlinear dynamical descriptors directly into the attention mechanism of LSTM-based autoencoders for unsupervised anomaly detection.

## 3. Materials and Methods

### 3.1. Dataset

Open source datasets play a critical role in the development and validation of PHM systems, particularly for rotating machinery components such as bearings. Bearings are essential elements that support and guide rotating shafts, and their degradation can be caused by operational stress, environmental conditions, or installation defects. These defects can lead to severe mechanical failures and unplanned downtime. Consequently, early fault detection is crucial for ensuring system reliability and reducing maintenance costs. Among various diagnostic approaches, vibration analysis has proven to be one of the most effective techniques for bearing fault detection, motivating the widespread use of publicly available vibration-based bearing datasets for studying early-stage mechanical anomalies [34].

#### 3.1.1. NASA Bearing Dataset

The NASA Bearing Dataset was introduced by the Center for Intelligent Maintenance Systems (IMS) at the University of Cincinnati and has become a benchmark dataset for bearing fault diagnosis and prognostics studies. The experimental setup consists of an AC motor, PCB 353B33 high-sensitivity accelerometers, and thermocouples. Four double-row bearings were mounted on a rotating shaft operating at a constant speed of 2000 rpm. An approximate radial load of 6000 lb (~27 kN) was applied to the shaft using a spring mechanism [35].

Vibration signals were recorded at 20 min intervals with a sampling frequency of 20 kHz, resulting in 20,480 data points per measurement. The data encompass four distinct operating states, consisting of normal operation and three fault scenarios associated with the inner race, outer race, and rolling element. In this study, only the data from Test 2 is used, comprising 984 samples and culminating in the natural development of an outer race fault in Bearing 1. This progressive degradation scenario makes the dataset particularly suitable for unsupervised anomaly detection and early fault identification tasks.

#### 3.1.2. CWRU Bearing Dataset

The CWRU bearing dataset was developed by the Case Western Reserve University Bearing Data Center and is one of the most widely used benchmark datasets for bearing fault diagnosis and anomaly detection studies [36]. The dataset contains vibration measurements acquired from a laboratory test rig under controlled operating conditions, with artificially induced bearing defects.

Faults were introduced using Electrical Discharge Machining (EDM) and include inner race, outer race, and ball defects with varying severities. Vibration signals were recorded from accelerometers mounted on the motor housing under different load conditions and rotational speeds [37,38]. In this study, the 48 kHz Drive-End (DE) vibration signals collected at approximately 1750 rpm were used as a benchmark dataset. The raw signals were segmented into fixed-length windows of 2048 samples, yielding a total of 2300 samples across normal and faulty operating conditions. This configuration has been widely adopted in the literature and provides a suitable testbed for evaluating unsupervised bearing anomaly detection performance [39]. Although the CWRU dataset is originally designed for fault classification, its controlled degradation scenarios make it suitable for benchmarking unsupervised anomaly detection methods.

#### 3.1.3. HUST Bearing Dataset

The HUST bearing dataset, provided by Hanoi University of Science and Technology, is a benchmark dataset designed for intelligent fault detection, condition monitoring, and PHM studies [39]. Compared to the CWRU dataset, HUST offers more realistic fault progression characteristics, as it contains naturally occurring bearing defects that evolve over time until failure, rather than artificially induced faults. This property makes the dataset particularly suitable for early fault detection, RUL estimation, and transfer learning research.

The dataset consists of raw vibration signals collected from ball bearings operating under multiple load conditions, with a sampling frequency of 51.2 kHz [40]. It includes normal conditions as well as inner race, outer race, and ball defects, along with their combined fault modes [39,40]. In this study, vibration data corresponding to the 6205-bearing type operating at 400 W were used. The signals were segmented into fixed-length windows of 1024 samples, resulting in 3500 samples across seven health conditions.

These characteristics make the HUST dataset well suited for assessing the robustness of the proposed unsupervised RQA-enhanced anomaly detection framework under realistic degradation scenarios.

### 3.2. Model

#### 3.2.1. Long-Short Term Memory (LSTM)

**Figure 2:**
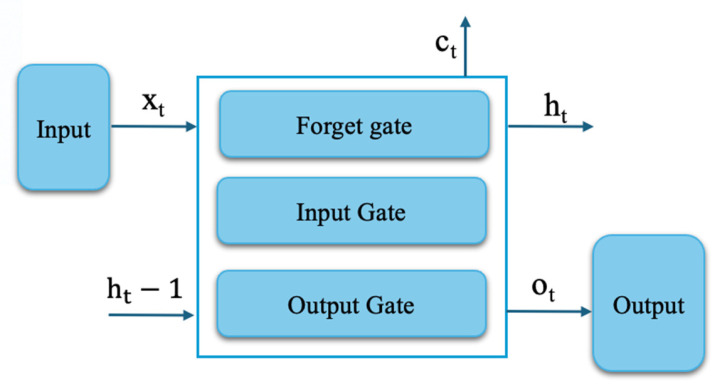
LSTM cell [42].

Long Short-Term Memory (LSTM) networks belong to the family of recurrent architectures developed to model sequential data by preserving long-range temporal information. This capability is achieved through an internal memory structure regulated by multiple gating units (Figure 2), which dynamically control information flow. Specifically, the input gate modulates the incorporation of new signals into the memory state, the forget gate adaptively attenuates obsolete information, and the output gate governs the propagation of the internal representation to subsequent layers [41].

Through its gated memory structure, the LSTM architecture supports long-term dependency modeling and stabilizes temporal learning dynamics. In contrast to standard RNNs, gradient degradation effects are alleviated by controlled information flow between successive cell states. The internal computations rely on sigmoid-based gating and tanh nonlinearities, enabling precise modulation of memory content at each time step, as formally described in prior work [43,44,45].

#### 3.2.2. Autoencoders (AE)

**Figure 3:**
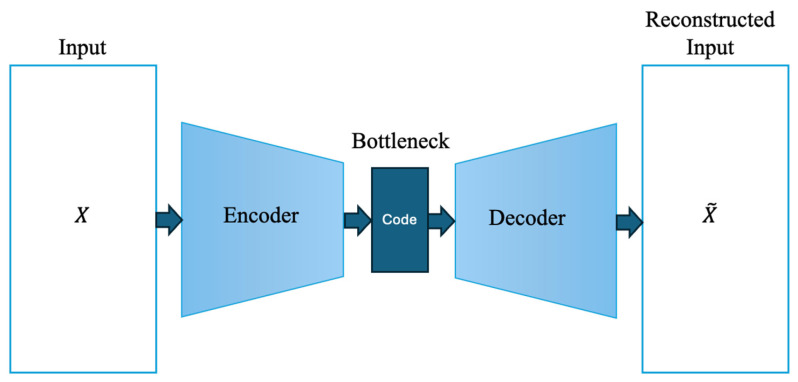
AE framework.

AE-based approaches are widely adopted for unsupervised anomaly detection in PHM due to their ability to model complex nonlinear relationships in multivariate sensor data without requiring fault labels. When trained exclusively on healthy data, AEs learn a compact latent representation of normal system behavior. Consequently, samples that deviate from this learned distribution are reconstructed with higher error, which can be directly exploited as an anomaly score [46]. The autoencoder consists of an encoder that compresses the input data and a decoder component that reconstructs the input from this representation (Figure 3).

An AE consists of an encoder that maps the input vector $x\in R^{n}$ into a lower-dimensional latent representation $z\in R^{m}$ (m<n), and a decoder that reconstructs the input from this representation. The encoder and decoder are defined as in Equations (1) and (2):

(1) $$z=f_{\theta }\left(x\right)=\sigma (W_{e}x+b_{e})$$

(2) $$\tilde{x}=g_{\emptyset }(z)=\sigma (W_{d}z+b_{d})$$

where $W_{e}$ and $W_{d}$ denote the encoder and decoder weight matrices, $b_{e}$ and $b_{d}$ denote the encoder and decoder bias vectors respectively, and $\sigma \left(.\right)$ is a nonlinear activation function. The reconstruction error is computed using Equation (3).

(3) $$L\left(x,\tilde{x}\right)={\left\|x-\tilde{x}\right\|}_{2}^{2}$$

The goal is to minimize the difference between the input and the reconstructed output. When detecting anomalies, the reconstruction error calculated for each sample is compared with the θ threshold value.

(4) $$L\left(x,\tilde{x}\right)>\theta$$

The threshold selection depends on the data distribution and application requirements and is commonly determined using statistical criteria such as percentiles or probabilistic distance measures, including kernel density estimation, Gaussian models, or Mahalanobis distance [47,48]. In this study, the reconstruction error serves as the primary anomaly indicator and provides the foundation for integrating RQA-enhanced attention mechanisms into the autoencoder framework.

#### 3.2.3. RQA Metrics

From a theoretical perspective, the concept of deterministic dynamical systems forms a foundational basis for recurrence-based analysis, particularly in nonlinear settings. This concept, first introduced for time series analysis by [49], describes system evolution as trajectories or orbits in a reconstructed phase space. Recurrence Plots (RP) were subsequently proposed to visualize and quantify the recurrence of system states by reconstructing the phase space from observed scalar time series data, resulting in a two-dimensional matrix representation of recurring patterns [50].

To reconstruct the underlying dynamical system, the time series is embedded into a higher-dimensional phase space using an appropriate embedding dimension m and delay parameter τ. The recurrence matrix is then obtained according to Equation (5) [8]:

(5) $$R_{i,j}=H\left(\varepsilon -\left\|x_{i}-x_{j}\right\|\right)=\left\{\begin{array}{c} 1,\varepsilon -\left\|x_{i}-x_{j}\right\|\geq 0\\ 0,\varepsilon -\left\|x_{i}-x_{j}\right\|<0 \end{array}\right.$$

In Equation (5), $R_{i,j}$ represents a binary recurrence matrix where $R_{i,j}=1$ indicates a recurrence (black point) between states $x_{i}$ and $x_{j}$, and $R_{i,j}=0$ indicates no recurrence (white point). H is the Heaviside function, ε is the recurrence threshold, and $\left\|.\right\|$ denotes the Euclidean norm. When plotted with *i* as the horizontal axis and *j* as the vertical axis, this binary matrix forms the RP.

The threshold ε is a critical parameter that determines the sensitivity of recurrence detection. Common approaches include: (1) fixed threshold based on a percentage of phase space diameter, (2) fixed recurrence rate, or (3) adaptive thresholds based on local density [51]. The choice depends on the data characteristics and analysis objectives.

In this study, RPs were computed directly from the scalar time series (embedding dimension *m* = 1, delay τ = 1), as RQA features were employed as structural descriptors rather than for attractor reconstruction. This choice avoids introducing additional hyperparameters and is sufficient when RQA is used for local dynamical characterization instead of invariant estimation. RPs were generated using a fixed recurrence rate strategy, where the distance threshold ε was automatically determined such that 20% of state pairs were recurrent. A relatively dense recurrence rate was preferred to ensure robust estimation of RQA statistics within short sliding windows. This approach ensures scale invariance and stabilizes RQA features across varying signal conditions. RQA metrics are computed independently for each time-windowed sample prior to attention computation, ensuring sample-wise dynamical characterization. For multivariate signals, channels are concatenated into a single 1D surrogate sequence prior to RP computation.

Although RPs facilitate qualitative assessment of dynamical systems, their interpretability is constrained by the absence of numerical descriptors. RQA overcomes this limitation by providing a set of metrics that quantify recurrence distributions and fine-scale diagonal features embedded in the RP structure [26,50].

RQA is a nonlinear mathematical and statistical technique used to extract meaningful features from time series, particularly in complex, chaotic, and non-stationary environments. Key RQA parameters such as recurrence rate, determinism, entropy, mean diagonal length, and trapping time help characterize fundamental dynamics and detect changes or anomalies in vibration signals [30,52,53]. Among these metrics, RR measures the recurrence rate of the time series. A high RR indicates that the system is more regular. Its mathematical representation is expressed as follows (Equation (6)).

(6) $$RR=\frac{1}{N^{2}}\sum_{i,j}^{N}R_{i,j}$$

In the equation, N is the total sample size. RR is the density measure of RP and represents the ratio of recurrence points to the total number of possible recurrences [54].

DET quantifies the proportion of recurrence points forming diagonal line structures, reflecting the presence of deterministic and predictable patterns in the system dynamics [55]. High DET values indicate more deterministic behavior as opposed to stochastic dynamics. DET is defined as shown in Equation (7),

(7) $$DET=\frac{\sum_{l=l_{min}}^{N}lP(l)}{\sum_{l=1}^{N}lP(l)}$$

In the equation, N represents the size of the RP matrix, l represents the length of the diagonal lines on the RP, and P(l) represents the frequency distribution of the diagonal line lengths. The minimum diagonal length is typically set to 2 to exclude tangential motion and single-point recurrences.

LAM quantifies the proportion of recurrence points that form vertical structures in the RP, indicating laminar states where the system dynamics remain relatively unchanged. High LAM values suggest the presence of intermittency or slowly varying regimes [55]. LAM is calculated as shown in Equation (8).

(8) $$LAM=\frac{\sum_{v=v_{min}}^{N}vP(v)}{\sum_{v=1}^{N}vP(v)}$$

v indicates the length of vertical lines formed by consecutive recurrence points on the RP. $v_{min}$ is the threshold for the shortest vertical line length to be considered. The minimum vertical line length $v_{min}$ is typically set to 2, as at least two consecutive recurrence points are required to define a line structure. $P\left(v\right)$ represents the frequency distribution of vertical lines with length v.

$L_{mean}$ represents the average length of the system’s recurring dynamic patterns. High $L_{mean}$ values indicate that the system has long-term correlations and high predictability, while low values indicate shorter-term and chaotic behavior (Equation (9)).

(9) $$L_{mean}=\frac{\sum_{l=l_{min}}^{N}lP(l)}{\sum_{l=l_{min}}^{N}P(l)}$$

In the equation, $P\left(l\right)$ is the number of diagonal lines with length l. $l_{min}$ is the minimum line length to be included in the calculation.

$L_{max}$, is one of the RQA metrics and represents the length of the longest diagonal line on the RP (Equation (10)). This metric is another indicator used to evaluate the deterministic (predictable) behavior of the system.

(10) $$L_{max}=max(\left\{l_{i};i=1,2,\ldots ,N\right\})$$

Here, $l_{i}$, is the length of the i-th diagonal line in RP.

ENTR measures the complexity of system dynamics based on the distribution of diagonal lines in RP (Equation (11)). It typically represents the Shannon entropy associated with the probability of detecting a diagonal line of full length l. High entropy values are associated with increased dynamical complexity, reflecting a broader distribution of diagonal line lengths, whereas low entropy values indicate the dominance of a limited number of characteristic time scales, corresponding to more regular or periodic system behavior [56].

(11) $$ENTR=-\sum_{l=min}^{N}P\left(l\right)lnP(l)$$

TT measures the average length of vertical lines in the RP, representing the mean time the system remains in a similar state (Equation (12)). High TT values indicate that the system tends to persist in certain states for extended periods, which may reflect stable operating conditions or, conversely, degraded states with reduced variability [26].

(12) $$TT=\frac{\sum_{v=v_{min}}^{N}vP(v)}{\sum_{v=v_{min}}^{N}P(v)}$$

In this study, the aim is to improve existing attention structures by incorporating chaotic metrics obtained using RQA. Within this scope, field-specific information about the physical processes that drive the system’s behavior has been obtained using recurrence-based features. As a result, the attention mechanism can now focus not only on raw temporal patterns but also on their derived features, potentially enhancing its ability to detect critical changes.

#### 3.2.4. RQA-Based Attention Mechanism

Adding RQA values to attention scores enables the model to include recurrence features when calculating attention weights, thereby helping the network focus on critical time steps where degradation accelerates. This significantly improves prediction accuracy, particularly in nonlinear systems.

For a given input tensor $X\in R^{B\times T\times F}$ the study examines three different RQA-Attention approaches: Hybrid QKVRQAA, CRQAA and ERQAA.

In the Hybrid QKVRQAA mechanism, which combines RQA pre-information with classical attention scores, the Query (*Q*), Key (*K*), and Value (*V*) of the classical attention mechanism are first calculated. Input data is passed through three linear projections to form the QKV matrices in sequence:

(13) $$Q=X.W_{Q}$$

(14) $$K=X.W_{K}$$

(15) $$V=X.W_{V}$$

In the equations, X is the input time series data, and $W_{Q},W_{K},W_{V}$ are the weight matrices. $b_{Q},b_{K},b_{V}$ are the model’s bias values. The scaled point-wise product score is calculated for each batch and time step as given in Equation (16).

(16) $$S=\frac{QK^{T}}{\sqrt{d}}$$

Each sample within the batch is converted into a single vector, and RP is generated from this vector. A 7-component RQA feature vector is obtained from RP and stacked into a tensor.

(17) $$x_{w}=vec\left(X_{w}\right)$$

(18) $${RP}_{w}=RecurrancePlot\left(x_{w}\right)\in {\left\{0,1\right\}}^{N\times N}$$

(19) $$r_{w}={\left[RR,DET,LAM,L_{mean},L_{max},ENTR,TT\right]}_{w}$$

(20) $$R=[r_{1};\ldots ;r_{W}]$$

Subsequently, an RQA-based channel weight vector is created.

(21) $$g=((\frac{1}{T}\sum_{t=1}^{T}x_{t}{)W}_{x})⨀tanh\left(RW_{r}\right)$$

In the equation, g is the channel weight vector, $x_{t}$ is the feature vector at time step t, $W_{x}$ is the input projection weight matrix, R is the RQA feature vector, and W is the RQA projection weight matrix. The tanh function normalizes the vector from RQA to the range [−1,1]. Element-wise multiplication (Hadamard product) is used to perform the channel-level multiplicative gating operation.

The channel weights are then reduced to a single scalar deviation coefficient and broadcast across time steps to obtain one global attention gate per batch:

(22) $$B_{rqa}=Broadcast\left(\tanh\left(gW_{\gamma }+b_{\gamma }\right)\right)$$

Here, $B_{rqa}$ functions as a global attention deviation term derived from channel-level fusion of RQA features. It is produced as a single scalar for each batch and propagated across all time steps. Consequently, adding $B_{rqa}$ o attention logits encourage the model to be sensitive not only to local temporal correlations but also to the overall nonlinear/chaotic structure of the series. $W_{\gamma }$ denotes the learnable weight vector associated with the global gate. Classical attention logits and the RQA-derived deviation are fused in a shared attention space:

(23) $$\tilde{S}=W_{1}S+W_{2}B_{rqa}$$

S denotes classical attention scores, $B_{rqa}$ denotes the RQA-based attention deviation, $W_{1}$ and $W_{2}$ are learnable fusion weights. This fusion map both components to a common scale and enables joint modeling of time-dependent relationships and recurrence-based dynamical complexity.

Finally, normalized attention weights are computed and applied to the value vectors in Equation (24):

(24) $$c=Softmax(\tilde{S})V$$

where c is the context representation. For each sample and time step, $\tilde{S}$ denotes the normalized fusion attention scores and V denotes the value vectors. The resulting context representation is passed to the decoder as an information-enriched feature representation.

Overall, unlike conventional attention mechanisms that rely solely on temporal similarity, the proposed formulation injects global nonlinear dynamical information as a structured bias term into attention scoring, improving robustness under noisy and non-stationary operating conditions.

In the second model, referred to as the CRQAA mechanism, RQA metrics are extracted directly from the input time series. This model is inspired by the work presented in [57], which suggests that attention mechanisms can be guided by physically interpretable features instead of relying solely on purely data-driven query–key interactions. Motivated by this idea, a physically informed channel-wise attention mechanism is proposed in this study, where RQA-based dynamical descriptors are used to modulate the attention representation through multiplicative interactions, rather than replacing the entire self-attention structure.

RQA metrics are computed for each input sample according to the formulations given in Equations (17)–(20). For each sample, seven RQA features are extracted from the corresponding recurrence plot, as defined in Equations (25) and (26). The resulting RQA feature vector $r_{w}$ is projected into a D-dimensional latent space using a fully connected layer with a hyperbolic tangent activation:

(25) $$z_{w}=tanh\left(r_{w}W_{r}+b_{r}\right)$$

(26) $$Z=[z_{1};\ldots ;z_{W}]$$

$W_{r}$ is the projection weight matrix, and $b_{r}$ is the bias vector. Z is formed by vertically stacking the $z_{w}$ vectors belonging to each sample. To fuse the RQA-based dynamic features with the input representation, the temporal average of the input time series $u_{w}$ is first computed. A channel-wise multiplicative interaction is then applied between the averaged input features and the projected RQA features in Equations (27) and (28).

(27) $$y_{w}=u_{w}⨀z_{w}$$

(28) $$Y=[y_{1};\ldots ;y_{B}]$$

Here, $u_{w}$ is the time average of the input time series. $z_{w}$ represents the chaotic-dynamic properties of the input. $y_{w}$ is the combined attention vector. The resulting vector $y_{w}$ constitutes a fused attention descriptor that integrates both signal content and dynamic complexity information. Through the Hadamard product, each channel becomes selectively sensitive to variations in both the input representation and the RQA-derived dynamical features, enabling a physically informed modulation of the attention mechanism.

The third mechanism, ERQAA, is identical to CRQAA, but it is calculated from the encoder’s hidden representation rather than the raw input signal. The encoder first compresses the input sequence into a lower-dimensional hidden space via two LSTM layers and a bottleneck dense layer. Subsequently, the hidden features are used to construct recurrence graphs and extract RQA metrics following Equations (17)–(20). All subsequent computations—projection (Equations (25) and (26)), channel-level gating (Equations (27) and (28))—remain identical to CRQAA. This variant test the hypothesis that encoder-based compression can either enhance or suppress chaotic signatures depending on the dataset characteristics. The most suitable variant (QKVRQAA/CRQAA/ERQAA) depends on sensor count, noise level, and signal complexity.

Finally, RQA descriptors are computed on fixed-length sliding windows to capture local, time-varying nonlinear dynamics relevant for early anomaly emergence. Computing RQA over the full sequence would result in a global summary and may dilute transient deviations; therefore, window-level RQA is adopted to align the dynamical prior with the anomaly scoring window. Each window is treated as an independent analysis unit, and anomaly scoring is performed only after the entire window has been observed. No samples beyond the window boundary are accessed during feature extraction or model inference, ensuring that the proposed formulation does not introduce information leakage from future time steps and preserves temporal causality at the window level.

### 3.3. Performance Evaluation Metrics

**Figure 4:**
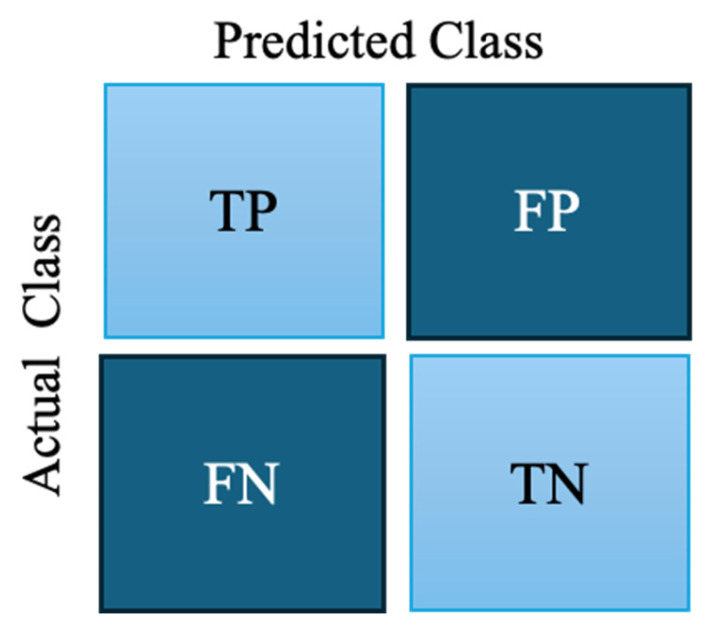
Confusion matrix for binary classification.

The effectiveness of the proposed anomaly detection framework is assessed using commonly adopted evaluation measures computed from the confusion matrix, which captures the distribution of correct and incorrect predictions [58,59]. In binary fault detection problems, where samples are categorized as normal or faulty, the confusion matrix consists of four fundamental components: True Positive (TP), True Negative (TN), False Positive (FP), and False Negative (FN), as illustrated in Figure 4.

Accuracy measures the proportion of correctly predicted instances relative to the entire dataset, as formulated in Equation (29).

(29) $$Accuracy=\frac{\left(TP+TN\right)}{\left(TP+TN+FP+FN\right)}$$

Although accuracy provides an overall performance indicator, it can be misleading in anomaly detection scenarios due to the inherently imbalanced nature of normal and faulty data distributions.

Precision and recall are therefore reported to better characterize model behavior under imbalance data. Precision measures the proportion of predicted faulty samples that are truly faulty (Equation (30)).

(30) $$Precision=\frac{TP}{\left(TP+FP\right)}$$

Recall (sensitivity) quantifies the proportion of actual faulty samples that are correctly detected as in Equation (31).

(31) $$Recall=\frac{TP}{\left(TP+FN\right)}$$

These metrics reflect complementary aspects of fault detection performance, where FP and FN may have different operational consequences. The F1-score is defined as the harmonic mean of precision and recall. This provides a balanced performance measure and is particularly suitable for imbalanced datasets (Equation (32)).

(32) $$F1-Score=2\times \frac{Precision\times Recall}{\left(Precision+Recall\right)}$$

In addition, the Receiver Operating Characteristic (ROC) curve and the Area Under the Curve (AUC) are used to assess the discriminative capability of the classifier across varying decision thresholds. The ROC curve plots the True Positive Rate (TPR) against the False Positive Rate (FPR) as in Equations (33) and (34).

(33) $$TPR=\frac{TP}{TP+FN}$$

(34) $$FPR=\frac{FP}{FP+TN}$$

The ROC–AUC score quantifies the probability that a randomly selected faulty sample is assigned a higher anomaly score than a randomly selected normal sample, making it a widely used evaluation metric for imbalanced classification problems [60]. Given the highly imbalanced nature of anomaly detection tasks, the F1-score and ROC–AUC are emphasized as the primary evaluation criteria in this study. In our implementation, ROC–AUC is computed based on reconstruction-error–derived anomaly scores obtained from the anomaly detection process, ensuring consistent and fair comparison across different models.

All experiments were conducted on a MacBook Pro with Apple M4 chip and 16 GB unified memory (Apple Inc., Cupertino, CA, USA) running macOS 15.6 (Apple Inc., Cupertino, CA, USA). The software stack comprised Python 3.11.0 (Python Software Foundation, Wilmington, DE, USA), TensorFlow 2.16.2 with Metal acceleration (Google LLC, Mountain View, CA, USA), NumPy 1.26.4 (NumFOCUS, Austin, TX, USA), and scikit-learn 1.6.1 (INRIA, Paris, France).

## 4. Results

**Figure 5:**
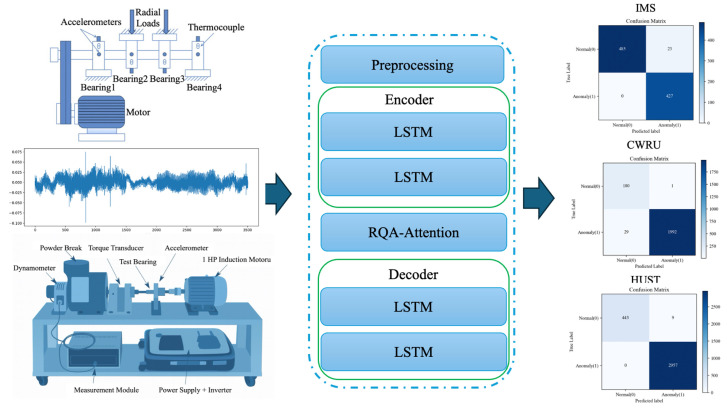
System Flow Chart.

In this study, an LSTM backbone was employed to capture temporal dependencies and extract sequential features. Within the anomaly detection framework, RQA-based attention mechanisms were integrated into an LSTM-AE architecture to enhance bearing anomaly detection performance. The proposed models differ according to the stage at which RQA information is incorporated: hybrid LSTM-AE-QKVRQAA combines RQA priors with QKV attention scores, LSTM-AE-CRQAA applies an RQA-Guided Channel Attention module at the input level, and the LSTM-AE-ERQAA introduces this guidance within the encoder’s latent representation. The overall system workflow is illustrated in Figure 5.

In the proposed model, each input sequence is first processed by the LSTM layers embedded within the encoder part of the AE, which capture the temporal dependencies across different time windows. Following the encoder, three distinct attention mechanisms are examined. The features enriched with RQA measures derived from RP are fed into a dedicated attention module. The RQA vector is incorporated as a gating bias into the attention scores, enabling the computation of attention weights that are sensitive to the regularity and recurrence properties of the underlying system dynamics.

**Figure 6:**
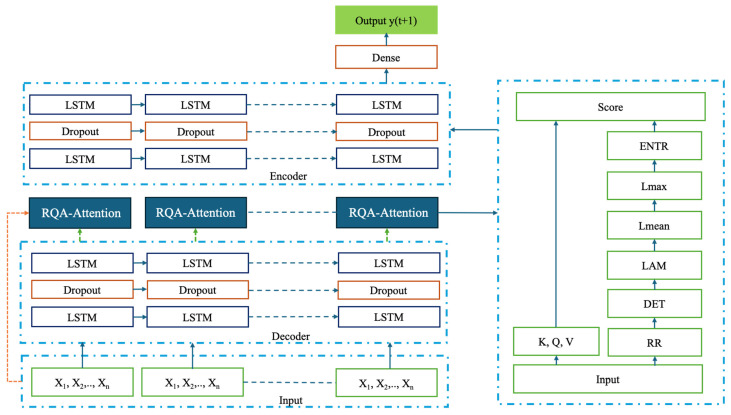
LSTM-AE-QKVRQAA Model Flow Chart.

The decoder LSTM layers then reconstruct the input sequence from this enriched representation, while anomaly detection is performed by comparing the reconstruction error against a threshold determined from the learned normal-condition distribution during training. Through this process, the RQA-enhanced attention mechanism assigns higher weights to patterns that reflect healthy operating behavior, thereby improving the model’s capability to detect bearing anomalies. A schematic illustration of the implemented models is presented in Figure 6.

**Figure 7:**
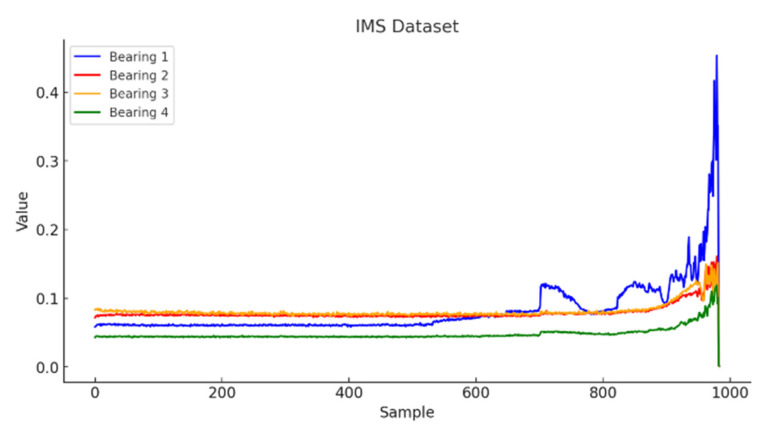
IMS dataset graph.

In this study, three publicly available bearing datasets commonly used in the literature were employed. A sample time-series visualization of the IMS dataset is presented in Figure 7. For the anomaly detection experiments, the first 531 samples of the signal were considered as healthy data, while the remaining portion was labeled as anomalous.

**Figure 8:**
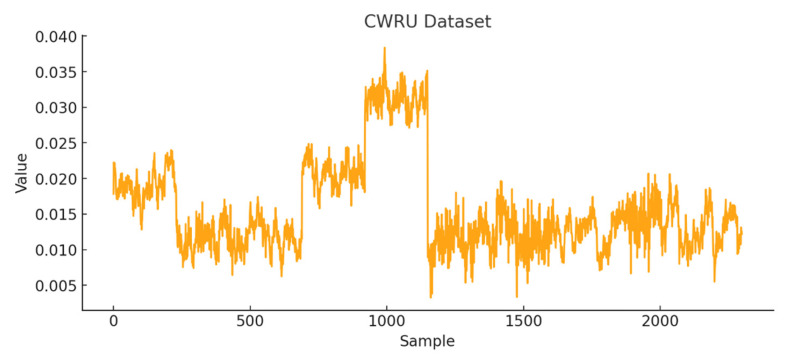
CWRU dataset graph.

A time-series representation of the CWRU dataset is provided in Figure 8. For the anomaly detection task, the first 230 samples were designated as healthy data, while the remaining portion of the signal was labeled as anomalous.

**Figure 9:**
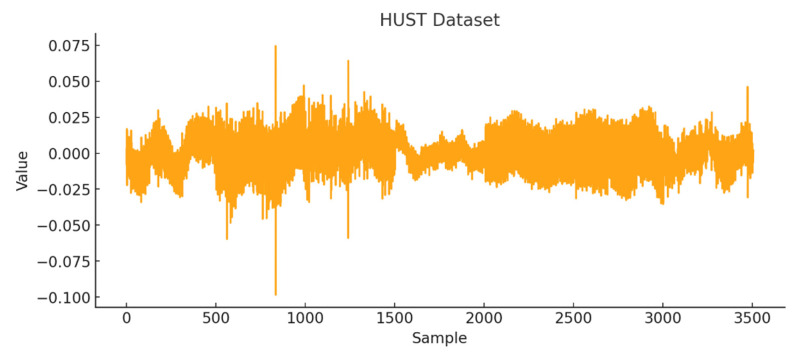
HUST dataset graph.

A time-series representation of the HUST dataset is shown in Figure 9. The segment corresponding to normal operation begins at sample 2004 and ends at sample 2504.

Windowed samples were split into training/validation/test sets using a time-ordered strategy to avoid leakage due to overlapping windows. The model was trained on healthy windows only; validation was performed on the remaining healthy portion, and test evaluation was conducted on the full timeline to assess detection performance across the entire degradation process.

As illustrated in the figures, the number of normal samples in both the CWRU and HUST datasets is considerably limited. Due to the scarcity of normal time-series segments and the presence of stochastic variations in sensor measurements that may negatively impact model performance, a noise-based data augmentation strategy was employed to increase the diversity of the training set. In this approach, each original sample was augmented by adding Gaussian noise with a zero mean and a predefined standard deviation (σ = noise level), generating new synthetic samples.

Mathematically, this process can be expressed as, ${\tilde{X}}^{\left(k\right)}=X+\eta^{\left(k\right)}$, where X denotes the original time series and $\eta^{\left(k\right)}∼N(0,\sigma^{2})$ represents the noise vector. In this study, the noise level was set to σ=0.01. The k noisy replicas produced from each sample were then combined with the original data, expanding the training set size to $\left(1+k\right)$ times its initial volume, with k=3 was selected. To validate the impact of Gaussian noise augmentation on recurrence-based features, a quantitative sensitivity analysis was conducted. As shown in Table 1, moderate noise levels (σ ≤ 0.05) preserve recurrence-driven anomaly separability, whereas excessive noise (σ = 0.1) degrades RQA-sensitive structures, leading to reduced detection performance. This confirms that the adopted percentile-based thresholding strategy ensures robustness under realistic noise levels, while also revealing its practical operating limits. This analysis also supports the design choice of percentile-based recurrence thresholding introduced in the methodology section.

This augmentation strategy improves the model’s robustness against measurement noise and environmental disturbances while reducing overfitting and enhancing generalization capability. Importantly, since recurrence plots were constructed using a percentile-based thresholding strategy, moderate noise levels (σ ≤ 0.05) do not significantly distort recurrence density, thereby ensuring consistent RQA-based feature extraction under realistic noise conditions.

To clarify the core concepts and procedural steps of the hybrid LSTM-AE-QKVRQAA model developed in this study, Algorithm 1 presents the fundamental pseudocode. The number of units in the LSTM layers and the dropout rates were optimized individually for each dataset (Table 2).
**Algorithm 1.** LSTM-AE-QKVRQAA**Input:**3D time series [sample, time, feature]**Training Parameters:**oEpoch: **100**oBatch: **16**oEarly stopping: **val_loss**, patience = 5**Output:**Trained **LSTM-AE-QKVRQAA** model**LSTM-AE-QKVRQAA model:**The input layer is definedThe LSTM layer is appliedLayer normalization is appliedDropout is appliedThe LSTM layer is applied againLayer normalization is appliedTime axis average is taken with GlobalAveragePooling1DBottleneck dense layer is appliedA Q-K-V projection is performed with dense layers.
oRP is extracted for each sample in the batchoRQA metrics are calculated:
▪RR▪DET▪LAM▪L_mean_▪L_max_▪ENTR▪TToRQA features are projected onto the dense layer dimensionoTemporal axis average and RQA vector are multiplicatively fused to produce a scalar gateoThis scalar gate is broadcast to all (T,T) positions and combined with classical scaled dot-product scoresoAttention weights are obtained using Softmax; they are multiplied by V and accumulated over time to produce the context vectorA channel-level weighted summary is obtainedIt is passed through the dense layer and ReLU activationIt is repeated for the time step using RepeatVector and passed to the decoderThe LSTM layer is appliedDropout is appliedThe LSTM layer is applied**Final layer: Dense layer, activation: linear**Adam optimizer, Learning rate: 0.001, and model is trained using the MSE loss function**LSTM-AE-QKVRQAA** model is returned

For multivariate time series like IMS dataset with 4 accelerometers, the signals were concatenated along the feature dimension before phase space reconstruction, resulting in a joint recurrence matrix that captures cross-channel temporal dependencies. This approach differs from computing separate RQA metrics for each channel and enables the attention mechanism to leverage inter-sensor correlations [61].

Table 2 presents the hyperparameters that are not commonly applied across all models.

Algorithm 2 provides the core pseudocode outlining the fundamental concepts and step-by-step procedure of the proposed LSTM-AE-CRQAA model.
**Algorithm 2.** LSTM-AE-CRQAA**Input:**3D time series data [sample, time, feature]**Training parameters:**oepoch: **100**obatch: **16**oEarly stopping: **val_loss**, patience = 5**Output:**Trained **LSTM-AE-CRQAA** model**LSTM-AE-CRQAA model:**The input layer is definedThe LSTM layer is appliedLayer normalization is appliedDropout is appliedThe LSTM layer is applied againLayer normalization is appliedTime axis average is taken with GlobalAveragePooling1DBottleneck dense layer is applied
oRP is extracted for each sample in the batchoRQA metrics are calculated:
▪RR▪DET▪LAM▪L_mean_▪L_max_▪ENTR▪TToThe RQA vector is projectedoThe input tensor is projectedo**A channel-level multiplicative gate is applied**
The time axis average is taken using the GlobalAveragePooling1D layerThe encoder summary is combined with channel weightsThe context vector is passed through a Dense layer and ReLU activationIt is repeated for the time step using RepeatVector and transferred to the decoderThe LSTM layer is appliedDropout is appliedThe LSTM layer is applied**Final layer: Dense layer, activation: linear**The model is trained using Adam optimization, learning rate: 0.001, and MSE loss function**LSTM-AE-CRQAA** model is returned

In the third model, LSTM-AE-ERQAA, the RQA metrics are computed from the encoder output rather than from the raw input sequence. All other computations and hyperparameter settings are kept identical to the LSTM-AE-CRQAA model. The objective of this design is to determine whether anomaly detection performance is more strongly influenced by RQA features derived directly from the input signal or by those computed from the latent representation produced by the encoder. To ensure full reproducibility, the random seeds were fixed at 42 for all experiments. The hyperparameters used in the model are summarized in Table 3.

In the threshold selection stage, the 3-sigma rule is employed. For each time window i, an anomaly score $s_{i}$ is computed, which corresponds to the reconstruction error of the autoencoder. Using the training set composed solely of fault-free samples, $D_{norm}={\left\{s_{i}\right\}}_{i=1}^{N}$, the location and scale parameters of the score distribution are estimated according to the following Equations (35) and (36):

(35) $$\hat{\mu }=\frac{1}{N}\sum_{i=1}^{N}s_{i}$$

(36) $$\hat{\sigma }=\sqrt{\frac{1}{N-1}\sum_{i=1}^{N}{\left(s_{i}-\hat{\mu }\right)}^{2}}$$

Since the reconstruction error in autoencoder-based anomaly detection does not generate negative anomalies, a one-sided 3-sigma rule was applied to capture only the extreme values on the upper side of the distribution. According to the one-sided 3-sigma rule, the decision threshold θ is computed in Equation (37):

(37) $$\theta =\hat{\mu }+k\hat{\sigma },\;\;\;\;k=3$$

For any new window, the decision function is defined in Equation (38):

(38) $$Anomaly\left(s\right)=\left\{\begin{array}{c} 1,s>\theta ,\\ 0,s\leq \theta . \end{array}\right.$$

The performance metrics of the models are presented in Table 4.

**Figure 10:**
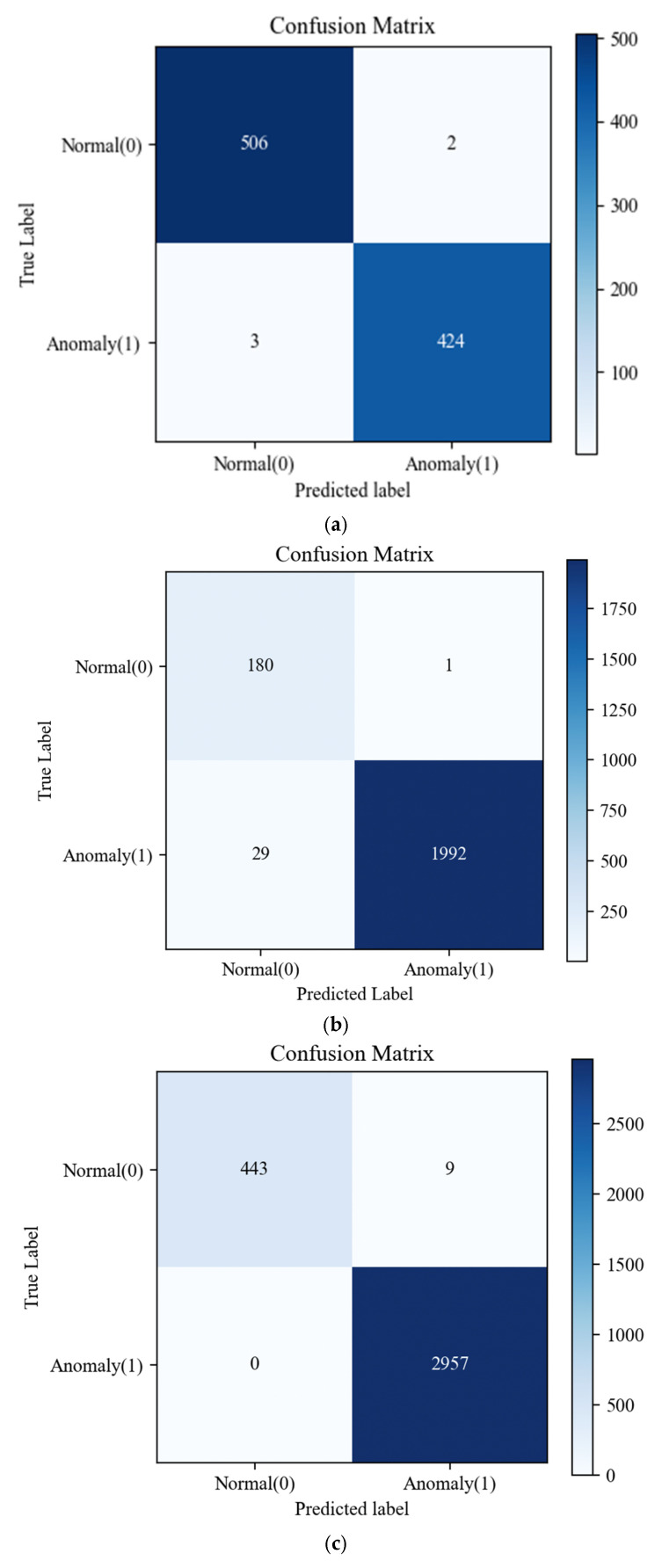
Confusion matrices of the datasets: (**a**) IMS, (**b**) CWRU, (**c**) HUST.

The confusion matrices of the best-performing models for the three datasets corresponding to the models yielding the highest F1-score and AUC on each dataset are visualized in Figure 10.

Table 4 summarizes the performance of the baseline LSTM-AE and the proposed RQA-guided attention models across the IMS, CWRU, and HUST datasets. Overall, the results indicate that incorporating RQA-derived dynamical descriptors into attention mechanisms consistently improves anomaly detection performance, with the gains becoming more pronounced as the dynamical complexity and noise level of the dataset increase. In the IMS dataset, which exhibits relatively regular and well-structured dynamics, the baseline LSTM-AE already achieves strong performance. Nevertheless, the proposed LSTM-AE-QKVRQAA model further improves the results, reaching an accuracy of 99.47%, an F1-score of 99.41%, and an AUC of 99.45%, indicating more balanced precision–recall behavior and improved sensitivity to subtle anomalies. This suggests that integrating RQA-informed dynamical priors into the QKV-based attention mechanism enhances temporal feature discrimination even in comparatively simple operating conditions. For the CWRU dataset, both LSTM-AE-QKVRQAA and LSTM-AE-CRQAA significantly outperform the baseline model by approximately 6–7% in terms of accuracy and F1-score, achieving near-perfect classification performance. These results highlight the effectiveness of RQA-derived nonlinear descriptors in guiding attention toward structurally meaningful recurrence patterns, which is particularly beneficial for distinguishing bearing fault conditions under varying operating loads. The most substantial improvement is observed on the HUST dataset, where the baseline LSTM-AE performs poorly due to strong noise and heterogeneous operating regimes. In contrast, RQA-guided models demonstrate a significant performance increase. Notably, the LSTM-AE-CRQAA model achieves an F1-score of 99.85% and an AUC of 99.00%, confirming the robustness of the proposed RQA-guided channel-attention mechanism under challenging and nonstationary conditions.

Overall, these findings demonstrate that the observed performance gains cannot be attributed solely to the backbone autoencoder architecture. Instead, they arise from the explicit incorporation of nonlinear dynamical information through RQA, which enriches feature representations, improves anomaly separability, and mitigates overfitting tendencies commonly observed in conventional attention-based models. Due to the superior performance achieved on the HUST dataset compared to existing approaches, an additional robustness analysis with respect to random seed initialization is conducted in this study. This analysis aims to evaluate the stability and reliability of the proposed RQA-guided attention mechanism beyond a single training run. The robustness results of the LSTM-AE-CRQAA model under different random seeds are summarized in Table 5.

As reported in Table 5, the proposed method demonstrates consistent robustness against random initialization effects. While performance varies across seeds, high detection capability is largely preserved. In particular, Seeds 24 and 42 yield near-perfect results, with F1-scores exceeding 99.7% and AUC values close to 99%, indicating excellent separability between normal and anomalous bearing conditions. Under less favorable initializations (e.g., SEED = 5 and SEED = 1024), the model still achieves competitive performance, with F1-scores of 83.6% and 97.7%, respectively. The SEED = 0 case exhibits a more conservative behavior characterized by perfect precision but reduced recall, indicating that random initialization primarily affects the operating decision threshold rather than the underlying feature representation. This behavior can be attributed to a tighter reconstruction error distribution, which leads to a higher effective threshold when statistical thresholding is applied, rather than to a degradation of the learned latent space. Importantly, an inspection of the learned parameter statistics across all seeds reveals stable weight distributions centered near zero with well-bounded standard deviations, indicating numerically stable training without pathological parameter growth. These observations confirm that the reported performance is not the result of a single favorable training instance but instead reflects the intrinsic modeling capacity of the proposed framework rather than a single favorable training run.

To further assess robustness across different datasets, the proposed LSTM-AE-QKVRQAA model was also evaluated on the IMS bearing dataset using the same set of random seeds (0, 5, 24, 42, and 1024). The corresponding results are summarized in Table 6.

As shown in Table 6, the proposed model demonstrates consistently strong performance across different random initializations. High recall values are maintained for all seeds, reaching or approaching 100% in several runs, which confirms reliable detection of anomalous bearing segments. While minor variations in precision and overall accuracy are observed due to stochastic training effects, the F1-score remains consistently high, ranging from approximately 96% to nearly 100%, and the AUC values consistently exceed 96%. The best overall performance is achieved under SEED = 1024, yielding an F1-score of 99.88% and an AUC of 99.90%. Importantly, none of the evaluated seeds results in a significant performance degradation, indicating that the effectiveness of the proposed approach is not dependent on a particular initialization. Moreover, the distributions of learned weights and biases remain highly consistent across different initializations, with parameter means centered near zero and comparable variance levels. This observation further confirms that the proposed RQA-guided attention mechanism is not overly sensitive to random initialization and exhibits robust and reproducible behavior across datasets with different dynamical characteristics. To ensure consistency and reproducibility across all experiments, a fixed random seed (SEED = 42) is used throughout the study for all datasets, unless otherwise stated.

**Figure 11:**
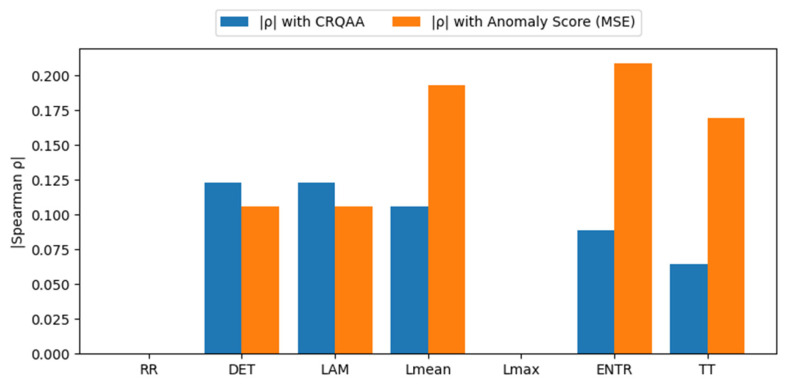
Impact of RQA metrics on LSTM-AE-CRQAA gating and anomaly score on the HUST dataset.

Figure 11 presents a Spearman rank correlation analysis between individual RQA metrics, the LSTM-AE-CRQAA gating magnitude, and the reconstruction-based anomaly score.

The results indicate that the proposed RQA-guided gating mechanism is primarily influenced by DET and LAM, suggesting that structurally repetitive and quasi-stationary dynamics play a dominant role in modulating the attention gate. This observation is consistent with the design objective of LSTM-AE-CRQAA, where recurrence structure is exploited as a dynamical prior rather than a direct anomaly indicator. In contrast, the anomaly score exhibits stronger associations with ENTR, TT, and L_mean_, reflecting increased dynamical complexity and disrupted recurrence patterns during anomalous operating conditions. These metrics capture variations in diagonal length distribution and temporal trapping behavior, which are known to increase under degradation or fault evolution. Notably, the RR remains nearly constant due to the adopted percentile-based thresholding strategy, explaining its negligible correlation with both the gating signal and the anomaly score. Overall, these findings demonstrate that CRQAA selectively leverages physically meaningful RQA descriptors instead of uniformly weighting all recurrence features.

**Figure 12:**
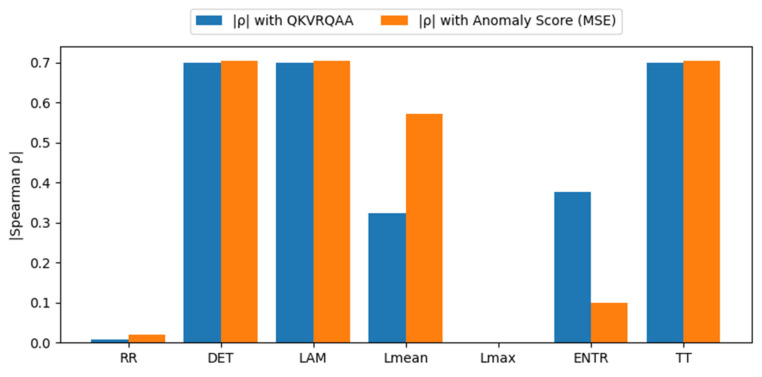
Impact of RQA metrics on LSTM-AE-QKVRQAA and anomaly score on the IMS dataset.

Figure 12 analyzes the relationship between individual RQA descriptors, the LSTM-AE-QKVRQAA gating strength, and the reconstruction-based anomaly score. LSTM-AE-QKVRQAA exhibits substantially stronger correlations, indicating a tighter coupling between recurrence dynamics and the attention modulation process.

DET, LAM, and TT show the strongest correlations with both the gating magnitude and the anomaly score, suggesting that LSTM-AE-QKVRQAA emphasizes persistent and structured dynamical patterns that are also reflected in reconstruction error. This behavior indicates that the fusion-based attention mechanism integrates recurrence structure more directly into the context representation. L_mean_ demonstrates a stronger association with anomaly magnitude than with the gating signal, implying a secondary role in severity estimation rather than attention control. In contrast, RR and L_max_ exhibit limited influence, which is expected given the percentile-based recurrence thresholding and the rarity of extreme diagonal structures in the analyzed signals. Overall, the results confirm that LSTM-AE-QKVRQAA preserves the interpretability of RQA-driven attention while strengthening the alignment between recurrence dynamics and anomaly severity.

Table 7 presents a sensitivity analysis of key RQA hyperparameters on the HUST dataset.

The results show that the proposed RQA-guided models are robust to moderate variations in embedding dimension and delay, confirming that RQA is primarily used as a structural descriptor rather than for precise attractor reconstruction. While a recurrence rate of 10% yields slightly higher performance on HUST, a fixed value of 20% was adopted throughout the main experiments to ensure consistency across datasets and to follow common practice in RQA-based studies. Notably, excessive recurrence density (30%) leads to a clear degradation in performance, indicating loss of discriminative recurrence structures.

## 5. Discussion

The performance of different RQA-aware attention architectures varies significantly across datasets due to their distinct signal characteristics. For the IMS dataset, which exhibits highly periodic dynamics, the LSTM-AE-CRQAA model shows limited temporal resolution, as channel-wise RQA aggregation reduces sensitivity to fine-grained phase variations in strongly periodic signals. In this case, the LSTM-AE-ERQAA model provides more stable results, as the encoder preserves dominant low-frequency periodic patterns sufficient for recurrence detection (F1-score = 93.68%). Nevertheless, the LSTM-AE-QKVRQAA model achieves the highest performance by jointly incorporating Q–K–V interactions and global RQA-derived deviation, benefiting from the stable dynamics that allow attention weights to be learned more clearly (F1-score = 99.41%).

For the CWRU dataset, the LSTM-AE-CRQAA model demonstrates superior performance, as RQA metrics computed directly from the input signal preserve more discriminative information in this high-noise environment. Applying RQA at the encoder output (LSTM-AE-ERQAA) leads to information loss, indicating that LSTM-AE-CRQAA is the most effective and lightweight approach for single-sensor systems with elevated noise levels.

The HUST bearing dataset [39] has been primarily utilized for supervised fault classification tasks [45,62]. However, unsupervised anomaly detection—critical for real-world scenarios where labeled failure data is scarce—remains underexplored for this dataset. This study addresses this gap by introducing an RQA-aware attention framework specifically designed for unsupervised anomaly detection on HUST bearing data. On this dataset, the LSTM-AE-CRQAA model achieves exceptional performance (F1-score = 99.85%), while LSTM-AE-ERQAA performs poorly (F1-score = 57.53%). This stark contrast reveals a critical insight: encoder-based dimensionality reduction suppresses high-frequency chaotic signatures essential for RQA in noise-dominant signals. Analysis of the latent representations shows that the HUST encoder (bottleneck dimension = 8) filters out high-frequency components to reduce noise. While beneficial for reconstruction, this low-pass filtering effect removes fine-grained dynamical structures (e.g., short diagonal lines in RP) that RQA relies on to distinguish chaotic from regular behavior.

The literature comparison with state-of-the-art methods is presented in Table 8.

While chaos theory and RQA have been extensively studied in bearing fault diagnosis, most existing work focuses on supervised fault classification rather than unsupervised anomaly detection. Several recent studies have explored unsupervised approaches on standard benchmark datasets, though with varying methodological frameworks and performance metrics. It should be noted that the comparison with prior studies is provided for contextual reference only, as differences in supervision level, preprocessing pipelines, and evaluation protocols prevent strict one-to-one benchmarking.

Studies employing RQA metrics for bearing fault diagnosis have predominantly focused on supervised classification frameworks. For example, experiments conducted on the CWRU bearing dataset using 12 kHz vibration recordings report anomaly detection accuracies reaching 96.97% [69].

DCC method [68] represents a strong supervised baseline reported in the recent literature, achieving 100% AUC, 100% accuracy, and 100% F1-score on the CWRU dataset (12 kHz). DCC employs a non-reconstructive approach with spectral normalization, directly scoring normality without autoencoder reconstruction. The proposed LSTM-AE-CRQAA achieves 99.25% F1-score and 99.01% AUC on CWRU. However, a direct comparison with the proposed approach is not straightforward, as these studies address supervised multi-class fault identification, whereas the present work focuses on unsupervised anomaly detection using higher-resolution 48 kHz signals, which preserve richer high-frequency fault-related dynamics. In addition, several aspects critical for fair benchmarking—such as class-wise performance, dataset imbalance handling, and evaluation protocols—are either not consistently reported or differ substantially across studies. Consequently, the reported results should be interpreted as complementary rather than directly comparable to the proposed framework.

Ref. [63] proposed AE-AnoWGAN, an unsupervised framework combining autoencoders with Wasserstein GANs for bearing anomaly detection. Raw vibration signals are transformed into time–frequency spectrograms via continuous wavelet transform and processed through a multi-encoder, multi-decoder GAN architecture. On the IMS dataset, the method achieved an AUC of 92.00%. However, the authors do not specify which of the three operating-condition subsets was used, limiting reproducibility. In comparison, the proposed LSTM-AE-QKVRQAA reaches higher performance (99.41% F1-score, 99.45% AUC) through joint Q–K–V attention integration.

Ref. [64] introduced MRRAE, combining convolutional autoencoders with memory modules that store prototypical normal patterns. The model detects anomalies by measuring deviations from stored memory representations, achieving 97.97% accuracy and 97.73% F1-score on the IMS dataset. While MRRAE effectively preserves representative patterns through memory augmentation, it lacks the temporal focusing capability inherent in QKV-based attention mechanisms. The proposed LSTM-AE-QKVRQAA, by contrast, dynamically recalibrates attention weights using RQA-derived chaos metrics, enabling real-time adaptation to evolving signal dynamics without fixed memory templates.

Addressing the scarcity or complete absence of fault samples, ref. [65] proposed DIDAD, a dual-stream CNN-based framework. Feature extractors process normal and test data separately, with outputs fused through an autoencoder-based module. Validated on the IMS dataset, DIDAD achieved accuracy exceeding 98.00%. The proposed LSTM-AE-QKVRQAA attains comparable accuracy (99.47% on IMS) while delivering more balanced performance across multiple metrics due to RQA-enhanced attention that captures both reconstruction error and dynamical complexity.

Ref. [66] introduced VCEAD, employing autoencoder-based reconstruction error alongside TCN-based vibration forecasting. Anomalies are detected using a variable cumulative error criterion. On the IMS dataset, VCEAD achieved 96.72% accuracy and 97.74% F1-score—performance closely matching LSTM-AE-QKVRQAA (99.41% F1-score). However, VCEAD relies on fixed threshold-based cumulative error, whereas the proposed RQA-aware attention provides adaptive anomaly scoring grounded in chaos-theoretic recurrence analysis, potentially offering better interpretability and robustness to non-stationary signals.

Ref. [67] proposed the DAAD framework, combining domain adaptation with unsupervised anomaly detection to address distribution shifts across operating conditions. On the CWRU dataset, DAAD achieved an AUC of 95.70%. Despite their effectiveness in cross-domain transfer via adversarial or distribution-based alignment, domain adaptation techniques conventionally depend on normal data from both source and target domains. In contrast, the proposed RQA-aware attention embeds chaos-theoretic invariants directly into the attention mechanism, enabling single-domain training while maintaining robustness to condition variations. On CWRU, LSTM-AE-CRQAA achieves 99.25% F1-score and 99.01% AUC without domain adaptation overhead.

All experiments in this study were conducted within individual datasets, following the commonly adopted evaluation protocol in unsupervised anomaly detection. While the proposed RQA-guided attention mechanism was validated on multiple bearing datasets exhibiting different dynamical characteristics (IMS, CWRU, and HUST), no explicit cross-dataset or domain-shift training–testing scenario was considered.

We acknowledge that such cross-dataset evaluation would provide stronger evidence regarding generalization under distributional shifts. However, in unsupervised anomaly detection, differences in sensor configuration, sampling frequency, operating conditions, and fault annotation standards across datasets often make direct cross-dataset transfer ill-posed without additional adaptation mechanisms. Investigating domain adaptation and cross-dataset generalization therefore constitutes an important direction for future work.

It should be noted that the reported correlations quantify association rather than causality; nevertheless, they provide useful insight into how different recurrence properties interact with the proposed attention mechanisms.

## 6. Conclusions

In this study, hybrid deep learning architectures were proposed to improve unsupervised bearing anomaly detection by systematically integrating recurrence quantification analysis (RQA) metrics into different stages of LSTM-based autoencoder models. RQA descriptors were embedded at the input level, encoder output, and within a QKV attention mechanism, resulting in three architectures: LSTM-AE-QKVRQAA, LSTM-AE-CRQAA, and LSTM-AE-ERQAA.

The proposed models were evaluated on three benchmark bearing datasets—IMS, CWRU, and HUST—characterized by different noise levels and dynamical behaviors. Experimental results demonstrate that RQA-enhanced attention mechanisms significantly improve anomaly detection performance by capturing nonlinear recurrence structures and temporal dependencies inherent in vibration signals. Among the proposed architectures, the hybrid LSTM-AE-QKVRQAA consistently achieved the most balanced and robust performance across datasets, highlighting the benefit of jointly modeling temporal attention and global RQA-based dynamical cues.

On the IMS dataset, LSTM-AE-QKVRQAA achieved a 99.41% F1-score and a 99.45% AUC, outperforming the baseline LSTM-AE. For the CWRU dataset, RQA-aware models improved accuracy and F1-score by approximately 6–7%, approaching near-perfect anomaly discrimination, with LSTM-AE-CRQAA achieving a 99.25% F1-score and a 99.01% AUC. In the more challenging HUST dataset, where the baseline model exhibited limited performance, the LSTM-AE-CRQAA architecture achieved an F1-score of 99.85% and an AUC of 99.00%, demonstrating strong robustness under noisy and heterogeneous operating conditions. Comparative analysis with state-of-the-art methods further confirms that LSTM-AE-QKVRQAA and LSTM-AE-CRQAA outperform existing deep learning-based anomaly detection approaches, particularly in terms of robustness across datasets with different noise levels and dynamical characteristics. These findings validate that embedding chaos-aware RQA descriptors into attention mechanisms provides an effective and principled way to model nonlinear dynamics, making the proposed framework well suited for practical PHM applications.

Despite the promising performance, several limitations should be acknowledged. First, RQA descriptors are computed using fixed embedding and recurrence parameters selected empirically, which may not optimally capture system dynamics under all operating conditions. Adaptive or data-driven parameter tuning could further improve robustness, especially in highly non-stationary environments. Second, the computation of recurrence plots and RQA metrics introduces additional computational cost compared to standard attention mechanisms. While acceptable for offline PHM analysis, this overhead may limit applicability in real-time or edge-based monitoring systems. In addition, the proposed models are trained in an offline manner and assume stationary degradation distributions. In realistic industrial settings, degradation patterns may evolve due to changing loads, environments, or maintenance actions. Incorporating online or continual learning strategies could help address such concept drift. Finally, this study focuses on univariate or globally aggregated multivariate recurrence analysis. Extending the framework to multi-scale and channel-wise RQA representations, as well as integrating physics-informed constraints, represents a promising direction for future research.

## Figures and Tables

**Table 1:** Effect of Gaussian noise augmentation on anomaly detection performance (HUST dataset).

Noise Std (σ)	F1-Score (%)	AUC (%)
0.10	52.67	67.87
0.05	98.40	98.24
0.01	99.85	99.00

**Table 2:** Dataset-Specific Hyperparameters.

Dataset	Hyperparameter	Value
IMS	LSTM Layer 1 Units	16
	LSTM Layer 2 Units	8
	Dropout Rate	0.2
	Attention Layer	8
	Dense Layer	8
CWRU	LSTM Layer 1 Units	64
	LSTM Layer 2 Units	32
	Dropout Rate	0.4
	Attention Layer	32
	Dense Layer	16
HUST	LSTM Layer 1 Units	128
	LSTM Layer 2 Units	64
	Dropout Rate	0.4
	Attention Layer	64

**Table 3:** Common Hyperparameters.

Hyperparameter	Value
Window Length	50
Step Size	1
Batch Size	16
Learning Rate	0.001
Optimizer	Adam
Maximum Epochs	100
Early Stopping	5
Validation Split	0.2

**Table 4:** Performance metrices.

Model	Dataset	Accuracy (%)	Precision (%)	Recall (%)	F1-Score (%)	AUC (%)
LSTM-AE	IMS	99.04	100	97.89	98.93	98.95
	CWRU	92.10	100	91.39	95.50	95.70
	HUST	20.36	100	8.18	15.13	54.09
LSTM-AE-QKVRQAA	IMS	99.47	99.53	99.30	99.41	99.45
	CWRU	97.05	100	96.78	98.37	97.05
	HUST	96.95	99.65	96.82	98.22	97.30
LSTM-AE-CRQAA	IMS	97.22	100	93.91	96.86	96.96
	CWRU	98.64	99.95	98.57	99.25	99.01
	HUST	99.74	99.70	100	99.85	99.00
LSTM-AE-ERQAA	IMS	94.55	99.47	88.52	93.68	94.07
	CWRU	98.59	99.75	98.71	99.23	97.98
	HUST	48.28	100	40.38	57.53	70.19

**Table 5:** Robustness analysis under different random seeds on the HUST dataset.

Random Seed	Accuracy (%)	Precision (%)	Recall (%)	F1-Score (%)	AUC (%)
0	48.05	100	40.11	57.25	70.05
5	75.48	99.72	71.93	83.58	85.30
24	99.47	99.70	99.70	99.70	98.85
42	99.74	99.70	100	99.85	99.00
1024	96.04	99.82	95.60	97.67	97.25

**Table 6:** Performance of the proposed LSTM-AE-QKVRQAA model on the IMS dataset under different random seeds.

Random Seed	Accuracy (%)	Precision (%)	Recall (%)	F1-Score (%)	AUC (%)
0	96.79	100	92.97	96.36	96.49
5	99.75	100	95.08	97.48	97.54
24	99.47	100	98.83	99.41	99.41
42	99.47	99.53	99.30	99.41	99.45
1024	99.89	99.77	1.0000	99.88	99.90

**Table 7:** Sensitivity analysis of RQA hyperparameters (recurrence rate, embedding dimension, and delay) on the HUST dataset.

Model	Dataset	Percentage	m	τ	Accuracy (%)	Precision (%)	Recall (%)	F1-Score (%)	AUC (%)
LSTM-AE-QKVRQAA	HUST	10	1	1	99.97	99.97	100	99.98	99.89
		20	1	1	96.95	99.65	96.82	98.22	97.30
		30	1	1	43.18	98.94	34.87	51.56	66.22
		20	2	1	99.94	99.93	100	99.97	99.78
		20	1	2	96.95	99.65	96.82	98.22	97.30
		20	2	2	99.88	99.86	100	99.93	99.56
LSTM-AE-CRQAA	HUST	10	1	1	99.97	99.97	100	99.98	99.98
		20	1	1	99.74	99.70	100	99.85	99.00
		30	1	1	84.78	99.80	82.62	90.40	90.76
		20	2	1	86.48	99.56	84.78	91.58	91.17
		20	1	2	99.74	99.70	100	99.85	99.00
		20	2	2	99.21	99.86	99.22	99.54	99.17

**Table 8:** Comparison with state-of-the-art methods on IMS, CWRU and HUST Dataset.

Model	Dataset	Accuracy (%)	F1-Score (%)	AUC (%)	Ref.
Auto-encoder Wasserstein Generative Adversarial Network (AE-AnoWGAN)	IMS	-	-	92.00	[63]
Memory Residual Regression Autoencoder (MRRAE)	IMS	97.97	97.73	-	[64]
Dual-Input Deep Anomaly Detection (DIDAD)	IMS	>98.00	-	-	[65]
Variable Cumulative Error anomaly Detection (VCEAD)	IMS	96.72	97.74		[66]
LSTM-AE-QKVRQAA	IMS	99.47	99.41	99.45	
Domain Adaptation-Based Anomaly Detection (DAAD)	CWRU	-	-	95.70	[67]
Deep Convolutional Critic (DCC, supervised)	CWRU	-	-	1.00	[68]
LSTM-AE-CRQAA	CWRU	98.64	99.25	99.01	
	HUST	99.74	99.85	99.00	

## Data Availability

This study is based on publicly available bearing fault datasets. The IMS dataset is obtained from the NASA Prognostics Data Repository, the CWRU dataset is provided by Case Western Reserve University, and the HUST dataset is released by Huazhong University of Science and Technology. All datasets are openly accessible, and no proprietary data were used.
